# *Bifidobacterium bifidum* G9‐1 Survives in the Intestinal Environment and Influences the Gut Microbiota Despite the Presence of Antimicrobials

**DOI:** 10.1111/1348-0421.13230

**Published:** 2025-06-29

**Authors:** Haruka Yokota, Yutaka Makizaki, Yoshiki Tanaka, Hiroshi Ohno

**Affiliations:** ^1^ Biofermin Pharmaceutical Co. Ltd. Kobe Japan

**Keywords:** antimicrobial, *Bifidobacterium bifidum* G9‐1 (BBG9‐1), intestinal microbiota, minimum inhibitory concentration, probiotic

## Abstract

Probiotics can help prevent antibiotic‐associated diarrhea; however, those lacking antimicrobial resistance may be ineffective during antimicrobial treatment. Here, we aimed to examine the effects of antimicrobials on the probiotic strain *Bifidobacterium bifidum* G9‐1 (BBG9‐1). Minimum inhibitory concentrations were determined in vitro by culturing *B. bifidum* G9‐1 with antimicrobials and assessing its viability. For in vivo analysis, germ‐free and specific pathogen‐free mice were administered *B. bifidum* G9‐1 along with antimicrobials. Gut microbiota composition and viable *B. bifidum* G9‐1 abundance were determined. *Bifidobacterium bifidum* G9‐1 was highly sensitive to antimicrobials in vitro. However, in a complex bacterial environment mimicking the gut environment, the abundance of viable *B. bifidum* G9‐1 was significantly high despite antimicrobial exposure. Dominant bacterial populations were more affected by antimicrobials than nondominant populations, with *B. bifidum* G9‐1 exhibiting increased viability in the presence of diverse bacterial species. *In vivo*, combined administration of antimicrobials and *B. bifidum* G9‐1 significantly reduced *B. bifidum* G9‐1 abundance in germ‐free mice but not in specific pathogen‐free mice, where the gut microbiota composition shifted following administration of *B. bifidum* G9‐1. The presence of diverse live bacteria in the intestine promotes the survival of *B. bifidum* G9‐1 and its beneficial effects, even in the presence of antimicrobials. This finding suggests that *B. bifidum* G9‐1, despite lacking intrinsic antimicrobial resistance, can survive and reach the large intestine, maintaining its probiotic function. Therefore, *B. bifidum* G9‐1 can potentially be used for antibiotic‐associated diarrhea prevention.

**Abbreviations**
AAD: antibiotic‐associated diarrhea
AMPC: amoxicillin
BBG9‐1: *Bifidobacterium bifidum* G9‐1
CAM: clarithromycin
CFDN: cefdinir
CFPN‐PI: cefcapene pivoxil
CFU: colony‐forming unit
FRPM: faropenem
GAM: Gifu anaerobic medium
GF: Germ‐free
IBS: irritable bowel syndrome
LVFX: levofloxacin
MIC: minimum inhibitory concentration
PBS: phosphate‐buffered saline
PMA: propidium monoazide
qPCR: quantitative polymerase chain reaction
SPF: specific pathogen‐free

## 1 Introduction

Antimicrobials are widely used to treat infectious diseases [1]. However, a common adverse effect is antibiotic‐associated diarrhea (AAD)—diarrhea associated with antimicrobial administration without a clear alternative etiology [2]. Intestinal bacterial imbalance contributes to AAD development [3, 4]. The use of probiotics is a promising strategy for AAD prevention [4, 5].

Probiotics offer various health benefits, including relief from constipation [6], diarrhea, irritable bowel syndrome (IBS), and inflammatory bowel disease [7], as well as eradication of *Helicobacter pylori*. *Bifidobacterium bifidum* G9‐1 (BBG9‐1), a probiotic strain, is an active ingredient in commercial prescription drugs for maintaining intestinal health, such as BIOFERMIN TABLETS and BIOFERMIN BIFIDUS POWDER (Biofermin Pharmaceutical Co. Ltd., Kobe, Japan). Studies have demonstrated its effectiveness in treating constipation [8, 9, 10], diarrhea [11], and IBS [12].

BBG9‐1 lacks antimicrobial resistance, and therefore, its effectiveness can be compromised when used in combination with antimicrobials. Minimum inhibitory concentration (MIC) is used to evaluate the antimicrobial susceptibility of bacteria in vitro. However, research suggests no correlation between MICs and in vivo efficacy [13]. Therefore, the effect of antimicrobials on probiotics in an in vivo gut environment cannot be extrapolated from MICs estimated in vitro, necessitating in vivo evaluations. Here, we aimed to investigate the effects of commonly used antimicrobials in clinical practice on BBG9‐1 viability both in vitro and in vivo.

## 2 Materials and Methods

### 2.1 Bacteria

BBG9‐1 (Biofermin Pharmaceutical Co. Ltd.) and *Bifidobacterium adolescentis* JCM 1275^T^ (Riken BRC, Tsukuba, Japan) were used as test and control strains, respectively. They were cultured in AccuDia Gifu anaerobic medium (GAM) broth (Shimadzu Diagnostics Corporation, Tokyo, Japan) supplemented with 0.7% glucose and 0.1% polysorbate 80 (culture medium) under anaerobic conditions at 37°C for 18 h, collected using centrifugation, and stored at −80°C until use.

The bacteria were resuspended in culture medium for in vitro studies. In addition to BBG9‐1, *Lactobacillus hamsteri* ID1255 (Biofermin Pharmaceutical Co. Ltd.) and Human Microbial Cell Cocktail Cell‐Mock‐003 (Cell‐Mock; NITE Bio Resource Center, Kisarazu, Japan) were included in in vitro experiments. *Lactobacillus hamsteri* was cultured and prepared in a manner similar to BBG9‐1. Cell‐Mock was suspended in culture medium before use. The configuration of Cell‐Mock is shown in Table S1. For in vivo studies, all strains were suspended in phosphate‐buffered saline (PBS; Nacalai Tesque Inc., Kyoto, Japan).

### 2.2 Antimicrobials

Antimicrobials commonly used in clinical practice were selected. Pure active ingredient of each antimicrobial was used for in vitro testing. The most common types of antimicrobials include amoxicillin trihydrate (AMPC; Fujifilm Wako Pure Chemical Corporation, Osaka, Japan), a penicillin; cefdinir (CFDN; Sigma‐Aldrich Co. LLC, St. Louis, MO, USA), a cephem; faropenem sodium hydrate (FRPM; Sigma‐Aldrich Co. LLC), a carbapenem; clarithromycin (CAM; Sigma‐Aldrich Co. LLC), a macrolide; and levofloxacin (LVFX; Sigma‐Aldrich Co. LLC), a new quinolone. AMPC, FRPM, and LVFX were dissolved in culture medium, and CFDN and CAM in 100 µL of dimethyl sulfoxide (Fujifilm Wako Pure Chemical Corporation) before being added to culture medium before their use.

In vivo studies were performed using commercially available formulations of the antimicrobials: amoxicillin (AMPC, Sawacillin Tablets; LTL Pharma Co. Ltd., Tokyo, Japan), cefcapene pivoxil (CFPN‐PI, Flomox Tablets; Shionogi & Co. Ltd., Osaka, Japan), faropenem (FRPM, Farom Tablets; Maruho Co. Ltd., Osaka, Japan), clarithromycin (CAM, Clarith Tablets; Taisho Pharmaceutical Co. Ltd., Tokyo, Japan), and levofloxacin (LVFX, Cravit Tablets; Daiichi Sankyo Co. Ltd., Tokyo, Japan). All antimicrobials were suspended in PBS before use.

### 2.3 Animals

Specific pathogen‐free (SPF) 6‐week‐old male Jcl:ICR mice (Clea Japan Inc., Tokyo, Japan) were housed at a stocking density of four mice per cage. For animal husbandry, we followed the methods described by Makizaki et al. [9]. All studies were conducted after approval by the Animal Experimentation Committee of Biofermin Pharmaceutical Co. Ltd. (Approval Numbers: 134‐008 and 137‐014). These procedures adhered to the Guide for the Care and Use of Laboratory Animals, Eighth Edition (Adthree Publishing Co. Ltd., Tokyo, Japan).

Germ‐free (GF) 6‐week‐old male MCH(ICR)/Jcl[Gf] mice (Clea Japan Inc.) were maintained in vinyl isolators (Clea Japan Inc.), at a stocking density of one animal per cage. For animal husbandry, we followed the methods described by Makizaki et al. [9]. All materials used in the vinyl isolators were sterilized by autoclaving at 127°C for 30 min.

### 2.4 In Vitro Study Design

#### 2.4.1 Antimicrobial Susceptibility Testing for *Bifidobacterium bifidum* G9‐1 (BBG9‐1)

The MIC of amoxicillin trihydrate (AMPC), cefdinir (CFDN), faropenem sodium hydrate (FRPM), clarithromycin (CAM), and levofloxacin (LVFX) were determined using the broth microdilution method, which is adopted as a standard method by the Japanese Society of Chemotherapy. BBG9‐1 and *Bifidobacterium adolescentis* were anaerobically cultured at 37°C for 18 h. MIC was defined as the minimum concentration of an antimicrobial at which BBG9‐1 and *B. adolescentis* growth (turbidity of the culture medium) was not visible after culture at 37°C for 24 h in culture medium containing each antimicrobial to achieve a bacterial count of approximately 10^6^ colony‐forming units (CFU)/well.

#### 2.4.2 Assessment of the Effect of Antimicrobials on BBG9‐1 Abundance in a Simulated Intestinal Environment

To mimic the intestinal environment, we used the feces of 5–6‐week‐old male SPF mice; the samples were collected and stored at −80°C immediately after defecation. The fecal samples were suspended in culture medium to prepare viable fecal suspensions. Additionally, culture medium alone was used to represent a sterile intestinal environment.

The effect of antimicrobials on viable BBG9‐1 abundance was estimated in the presence of fecal suspension at different concentrations. Fecal suspensions of three different concentrations (0, 1, and 10 mg/mL) were spiked with BBG9‐1 and antimicrobials and cultured anaerobically at 37°C for 24 h. Culture medium was used instead of fecal suspension for the 0 mg/mL condition.

We investigated BBG9‐1 viability under different conditions of fecal suspension in the presence of antimicrobials: culture medium alone, fecal suspension in which the intestinal bacteria were killed by high‐pressure steam sterilization (121°C, 20 min) (sterilized feces), and viable fecal suspension (feces). All tests had a starting BBG9‐1 concentration of 1.0 × 10^6^ CFU/mL and a final antimicrobial concentration of twice the MIC (Table 1) previously estimated for BBG9‐1 (0.25 µg/mL for AMPC, FRPM, and CAM; 4.0 µg/mL for CFDN; 8.0 µg/mL for LVFX). After culturing, viable counts of BBG9‐1 in the culture medium were determined using propidium monoazide quantitative polymerase chain reaction (PMA‐qPCR) with BBG9‐1‐specific primers.

**Table 1:** Minimum inhibitory concentration (MIC) of antimicrobials tested against *Bifidobacterium bifidum* G9‐1 (BBG9‐1) and *Bifidobacterium adolescentis*.

Bacterium	MIC (μg/mL)				
	AMPC	CFDN	FRPM	CAM	LVFX
BBG9‐1	≦ 0.125	2.0	≦ 0.125	≦ 0.125	4.0
*B. adolescentis*	0.25	0.25	0.25	≦ 0.125	4.0

Abbreviations: AMPC, amoxicillin; BBG9‐1, *Bifidobacterium bifidum* G9‐1; *B. adolescentis*, *Bifidobacterium adolescentis* JCM 1275^T^; CAM, clarithromycin; CFDN, cefdinir; FRPM, faropenem; LVFX, levofloxacin.

#### 2.4.3 Effect of Antimicrobials on Dominant and Nondominant Bacteria

The viability of *Lactobacillus*, the dominant bacterium, and *Bacteroides*, the nondominant bacterium, in mouse feces was examined in the presence of antimicrobials. AMPC, CFDN, FRPM, CAM, and LVFX were added to 10 mg/mL fecal suspension, and the samples were cultured anaerobically at 37°C for 24 h. Culture medium alone was used for the group with no antimicrobials (0 µg/mL). To clearly determine the effect of each antimicrobial, the final concentrations were set higher than their respective MIC: 64 µg/mL for AMPC, CFDN, FRPM, and CAM, and 128 µg/mL for LVFX. After culture, the viable count of *Lactobacillus* and *Bacteroides* in the culture medium was determined using PMA‐qPCR with primers specific for each genus.

#### 2.4.4 Effect of Antimicrobials on *Lactobacillus* and BBG9‐1 Abundance

The viability of *Lactobacillus* and BBG9‐1 in the presence of antimicrobials was determined in a 100:1 Coculture of *Lactobacillus* and BBG9‐1. *Lactobacillus hamsteri*, BBG9‐1, and various antimicrobials were added to the culture medium and cultured anaerobically at 37°C for 24 h. To clearly determine the effect of each antimicrobial, the final concentrations were set higher than their respective MIC values: 64 µg/mL for AMPC, CFDN, FRPM, and CAM, and 128 µg/mL for LVFX. Culture media were inoculated with 1.0 × 10^6^ CFU/ml *L. hamsteri* and 1.0 × 10^4^ CFU/mL BBG9‐1. Subsequently, the viable counts of *L. hamsteri* and BBG9‐1 were determined using PMA‐qPCR with *L. hamsteri‐* and BBG9‐1‐specific primers.

#### 2.4.5 Assessment of the Effect of Antimicrobials on BBG9‐1 Abundance in Co‐Cultures With Other Bacteria

BBG9‐1 viability was compared in co‐cultures containing various bacterial species at different abundance levels in the presence of antimicrobials. BBG9‐1, Cell‐Mock (Table S1), and various antimicrobials (64 µg/mL for AMPC, FRPM, CAM, and CFDN; 128 µg/mL for LVFX) were added to culture medium and cocultured anaerobically at 37°C for 24 h. The starting concentration of BBG9‐1 was 1.0 × 10^6^ CFU/mL and that of Cell‐Mock varied from 1.0 × 10^6^ to 10^9^ CFU/mL. After culture, viable counts of BBG9‐1 in the culture medium were determined using PMA‐qPCR with BBG9‐1‐specific primers.

### 2.5 In Vivo Study Design

#### 2.5.1 Effect of Each Antimicrobial on the Viability of BBG9‐1 in Germ‐Free (GF) Mice

GF mice were treated with BBG9‐1 alone in PBS or BBG9‐1 + antimicrobials (*n* = 3 per group) for three consecutive days. BBG9‐1 was administered at 1.0 × 10^9^ CFU/mouse three times daily. The doses of each antimicrobial were as follows: each was administered to mice at a dose equivalent to the approved daily dose for a 60 kg adult in Japan. AMPC at 4.2 mg/kg, CFPN‐PI at 1.7 mg/kg, and FRPM at 3.3 mg/kg were administered three times daily; CAM at 3.3 mg/kg was administered twice daily; and LVFX at 8.3 mg/kg was administered once daily. Feces were collected a day after the last dose, and viable counts of BBG9‐1 in the feces were determined using the agar plate dilution method. This study was conducted after approval by the Animal Experimentation Committee of Biofermin Pharmaceutical Co. Ltd. (Approval Number: 134‐008). The procedures adhered to the Guide for the Care and Use of Laboratory Animals, Eighth Edition (Adthree Publishing Co. Ltd., Tokyo, Japan).

#### 2.5.2 Assessment of the Effects of Each Antimicrobial on BBG9‐1 Viability and Gut Microbiota in SPF Mice

SPF mice were divided into three groups (*n* = 8/group) to assess the effects of each antimicrobial on BBG9‐1 viability and gut microbiota composition. The vehicle group received PBS alone, the antimicrobial alone group received antimicrobial and PBS, and the BBG9‐1 + antimicrobial group received BBG9‐1 and the designated antimicrobial. This study was conducted after approval by the Animal Experimentation Committee of Biofermin Pharmaceutical Co. Ltd. (Approval Number: 137‐014). The procedures adhered to the Guide for the Care and Use of Laboratory Animals, Eighth Edition (Adthree Publishing Co. Ltd., Tokyo, Japan).

SPF mice were treated with PBS, BBG9‐1, or BBG9‐1 + antimicrobial (*n* = 8/group) for three consecutive days. BBG9‐1 was administered at 1.0 × 10^9^ CFU/mouse three times daily. The doses of each antimicrobial were as follows: each was administered to mice at a dose equivalent to the approved daily dose for a 60 kg adult in Japan. AMPC at 4.2 mg/kg, CFPN‐PI at 1.7 mg/kg, and FRPM at 3.3 mg/kg were administered three times daily; CAM at 3.3 mg/kg was administered twice daily; and LVFX at 8.3 mg/kg was administered once daily. Fecal samples were collected and stored at −80°C 1 day after the final dose for the analysis of viable BBG9‐1 abundance in the feces using PMA‐qPCR and for gut microbiota analysis. This study was conducted after approval by the Animal Experimentation Committee of Biofermin Pharmaceutical Co. Ltd. (Approval Number: 134‐008). The procedures adhered to the Guide for the Care and Use of Laboratory Animals, Eighth Edition (Adthree Publishing Co. Ltd., Tokyo, Japan).

#### 2.5.3 Quantification of Viable Bacteria Using the Agar Plate Dilution Method

In the GF mice study, fecal samples were serially diluted 10‐fold in 1 L of purified water containing 6.0 g K_2_HPO_4_, 4.5 g KH_2_PO_4_, 0.5 g polysorbate 80, 0.5 g l‐cysteine HCl, and 1.0 g agar. Subsequently, 50 µL of each dilution was plated on AccuDia GAM agar plates (Shimadzu Diagnostics Corporation, Tokyo, Japan) supplemented with 0.7% glucose and 0.1% polysorbate 80, and then cultured anaerobically at 37°C for 48 h. Viable counts of BBG9‐1 were calculated based on the number of colonies that developed.

#### 2.5.4 Determination of Viable Bacterial Counts Using PMA‐qPCR

In the in vitro experiments, following culture, the culture medium was centrifuged at 14,000*g* for 5 min to collect the cell pellets. In in vivo experiments, approximately 20 mg of fecal samples was used. Samples from both in vitro and in vivo studies were washed three times with PBS before resuspension in 200 µL of PBS. PMA (Biotium Inc., Fremont, CA, USA) treatment was performed following a previously reported method [14]. DNA was extracted from the pellets using the bead‐phenol method [15]. qPCR was performed using the 2 × TaqMan Fast Advanced Master Mix (Thermo Fisher Scientific Inc., Waltham, MA, USA) and Applied Biosystems QuantStudio 3 Real‐Time PCR System (Thermo Fisher Scientific Inc.). The following primers and probe were used for amplification: forward primer 5′‐GTTCTAGCTGTTAGACCCATTT‐3′, reverse primer 5′‐GAATGACTTCCGTCTTGAAC‐3′, and probe 5′‐TGCATCCCTTTGCTCATATCTTAGAGTTGA‐3′. The amplification conditions were as follows: 50°C for 2 min, 95°C for 2 min, and 40 cycles at 95°C for 1 s and 60°C for 20 s. Each sample was analyzed in duplicate.

#### 2.5.5 Gut Microbiota Analysis

Gut microbiota composition was assessed using in‐depth 16S rRNA sequencing. DNA was extracted from feces using the bead–phenol method [15], and the V3–V4 region of the 16S rRNA gene was amplified and sequenced on the Illumina MiSeq platform (Illumina, San Diego, CA, USA) [16]. The sequencing data were analyzed using the QIIME2 pipeline [17], version 2021.4 (https://qiime2.org). β‐Diversity analysis, which explores the overall microbiota composition between samples, was performed using QIIME2. Furthermore, to enable comparisons within each antimicrobial group, analyses were performed using a distance metric appropriate for the specific antimicrobial under investigation: unweighted UniFrac (for AMPC), Bray–Curtis (for CFPN‐PI and FRPM), Jaccard (for CAM), and standardized Euclidean distances (for LVFX). Principal coordinate analysis was performed using QIIME2, and dissimilarities between samples based on the distance data obtained were visualized using the ggplot2 package in R (version 4.1.1).

### 2.6 Statistical Analysis

Data from the in vitro studies were compared between groups using the Tukey–Kramer or Steel method. Welch's *t*‐test was used to compare data between two groups. For in vivo study data, Dunnett's test was used. JMP statistical software (SAS Institute Inc., Cary, NC, USA) was used for statistical analysis. For the gut microbiota analysis, permutational analysis of variance was used to analyze β‐diversity. For multiple comparisons, the false discovery rate was controlled using Benjamini–Hochberg adjustment. Results with *p*‐value < 0.05 were considered significant.

## 3 Results

### 3.1 Antimicrobial Susceptibility of BBG9‐1

To determine the antimicrobial susceptibility of BBG9‐1, the MIC was evaluated. The MIC values of each antimicrobial for BBG9‐1 were as follows: 0.125 µg/mL or lower for AMPC, FRPM, and CAM; 2.0 µg/mL for CFDN; and 4.0 µg/mL for LVFX (Table 1). For the control *B. adolescentis*, the MIC values were as follows: 0.25 µg/mL for AMPC, CFDN, and FRPM; 0.125 µg/mL or less for CAM; and 4.0 µg/mL for LVFX (Table 1). BBG9‐1 exhibited susceptibility to the tested antimicrobials.

### 3.2 Effect of Antimicrobials on BBG9‐1 Abundance in In Vitro Coculture With Fecal Suspensions

**Figure 1:**
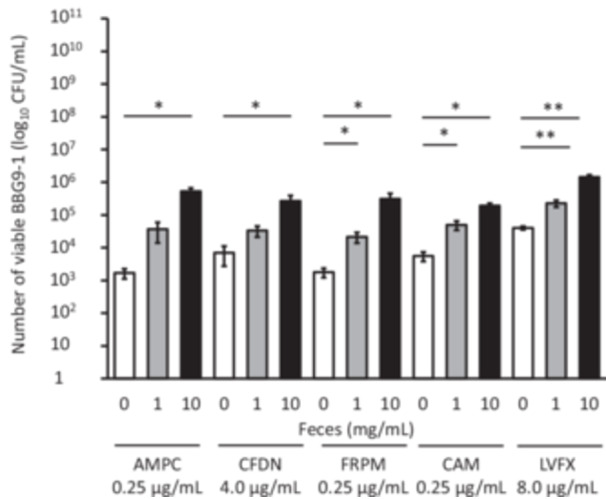
Effect of various antimicrobials on BBG9‐1 viability in specific pathogen‐free (SPF) mouse feces. Data (*n* = 4–6) are presented as mean ± standard error of the mean (SE). **p < 0.01, *p < 0.05 by Steel test. AMPC, amoxicillin; BBG9‐1, *Bifidobacterium bifidum* G9‐1; CAM, clarithromycin; CFDN, cefdinir; CFU, colony‐forming unit; FRPM, faropenem; LVFX, levofloxacin.

First, we investigated the effect of the presence of fecal suspension on the viability of BBG9‐1. Under 1 mg/mL fecal suspension condition, the viable counts of BBG9‐1 were significantly higher in the BBG9‐1 + FRPM, CAM, and LVFX groups than in groups without fecal suspension. Additionally, under 10 mg/ml fecal suspension condition, the viable counts of BBG9‐1 were significantly higher in all BBG9‐1 + antimicrobial groups than in their counterparts without fecal suspension (Figure 1).

**Figure 2:**
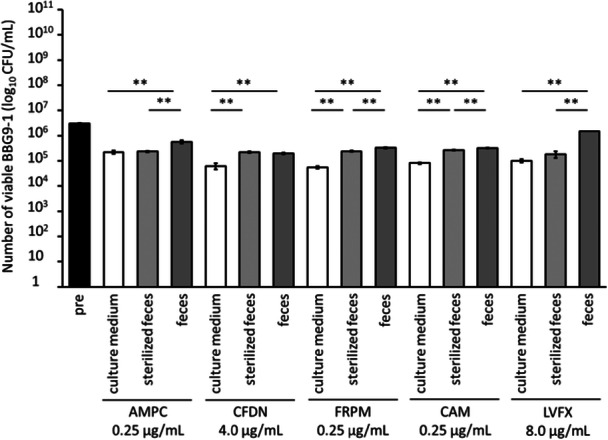
Viable counts of BBG9‐1 in the presence of SPF mouse feces. Data (*n* = 4) are presented as mean ± SE. **p < 0.01 by Tukey–Kramer test. AMPC, amoxicillin; BBG9‐1, *Bifidobacterium bifidum* G9‐1; CAM, clarithromycin; CFDN, cefdinir; CFU, colony‐forming unit; FRPM, faropenem; LVFX, levofloxacin; pre, before culture. [Correction added on 30 July 2025, after first online publication: The figure has been revised. The term “modified GAM” has been corrected to “culture medium”.]

Next, we evaluated whether the viability of BBG9‐1 was affected by whether the bacteria in the fecal suspension were alive or killed. In the BBG9‐1 + AMPC and LVFX groups, the viable counts of BBG9‐1 were significantly higher in cultures containing live bacteria (feces group) than in the culture medium. The feces group also exhibited significantly higher viable counts of BBG9‐1 than the counterparts with dead bacteria (sterilized feces group). In the BBG9‐1 + CFDN group, the viable counts of BBG9‐1 were significantly higher in the sterilized feces and feces groups than in the culture medium group. The same trend was observed in the BBG9‐1 + FRPM and CAM groups; additionally, the feces group had significantly higher counts of BBG9‐1 than the sterilized feces group (Figure 2). These results suggest that live intestinal bacteria facilitated the survival of BBG9‐1 in the presence of antimicrobials.

### 3.3 Effect of Antimicrobials on the Abundance of Dominant and Nondominant Bacteria in In Vitro Coculture

**Figure 3:**
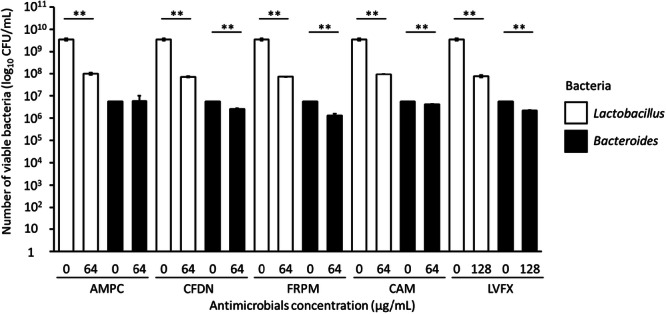
Effect of various antimicrobials on the viable counts of *Lactobacillus* and *Bacteroides* in specific pathogen‐free (SPF) mouse feces. Data (*n* = 3) are presented as mean ± standard error of the mean (SE). **p < 0.01 by Welch's *t‐*test. AMPC, amoxicillin; CAM, clarithromycin; CFDN, cefdinir; CFU, colony‐forming unit; FRPM, faropenem; LVFX, levofloxacin.

We evaluated whether the dominant bacterium *Lactobacillus* or the nondominant bacterium *Bacteroides* in feces were more affected by antimicrobial treatment. The viable counts of *Lactobacillus* were significantly lower in the AMPC‐treated group than in the untreated group. The viable counts of *Lactobacillus* and *Bacteroides* were significantly lower in the CFDN‐, FRPM‐, CAM‐, and LVFX‐treated groups than in the untreated groups. Additionally, for all antimicrobials, the reduction in the counts of *Lactobacillus* was greater than those for *Bacteroides* (Figure 3), The dominant bacterium was more affected by the antimicrobials.

### 3.4 Effect of Antimicrobials on the Abundance of *L. Hamsteri* and BBG9‐1 in In Vitro Coculture

**Figure 4:**
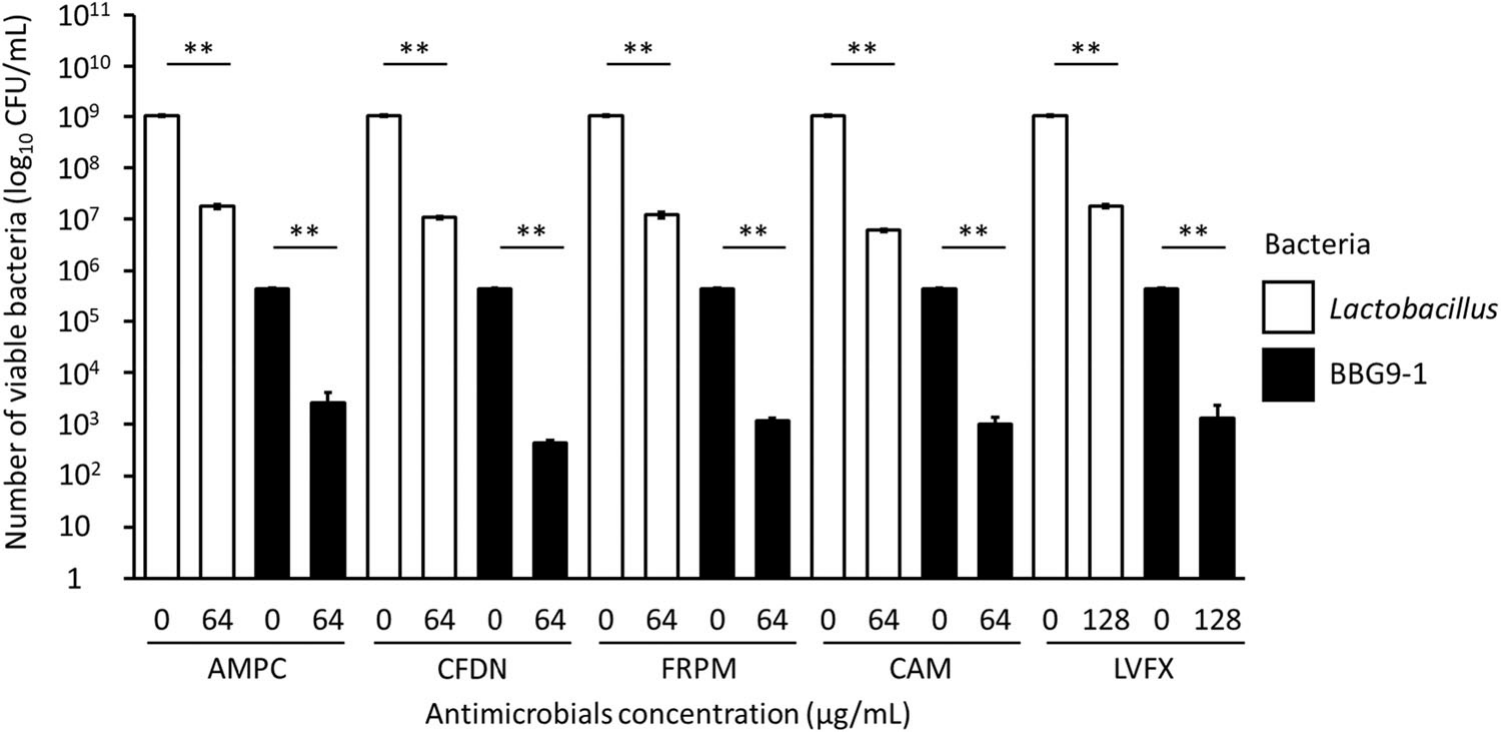
Effect of various antimicrobials on the viable counts of *Lactobacillus* and BBG9‐1. Data (*n* = 3) are presented as mean ± SE. **p < 0.01 by Welch's *t‐*test. AMPC, amoxicillin; BBG9‐1, *Bifidobacterium bifidum* G9‐1; CAM, clarithromycin; CFDN, cefdinir; CFU, colony‐forming unit; FRPM, faropenem; LVFX, levofloxacin.

As the dominant bacterium was more susceptible to antimicrobial treatment (Figure 3), we cocultured BBG9‐1 with the dominant bacterium Lactobacillus to evaluate its viability.　The counts of *L. hamsteri* and BBG9‐1 were significantly lower in all antimicrobial‐treated groups than in the untreated groups (Figure 4). This result indicates that BBG9‐1 is affected by antimicrobials when only one dominant species is present.

### 3.5 Effect of Antimicrobials on BBG9‐1 Abundance in In Vitro Co‐Cultures

**Figure 5:**
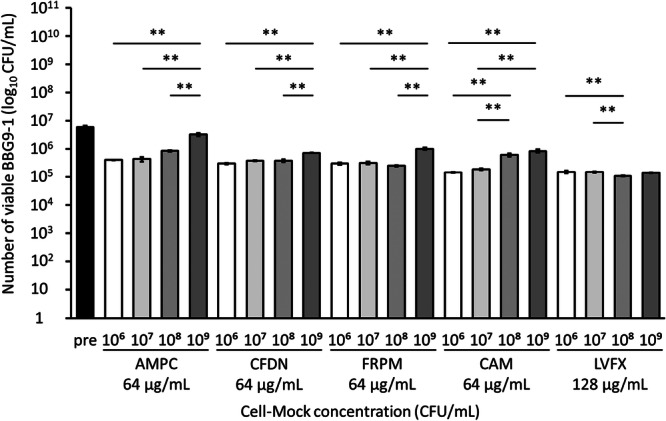
Viable counts of BBG9‐1 with various antimicrobials and Cell‐Mock at different concentrations (10^6^–10^9^ CFU/mL). Data (*n* = 3) are presented as mean ± SE. **p < 0.01 by Tukey–Kramer test. AMPC, amoxicillin; BBG9‐1, *Bifidobacterium bifidum* G9‐1; CAM, clarithromycin; CFDN, cefdinir; CFU, colony‐forming unit; FRPM, faropenem; LVFX, levofloxacin; pre, before culture.

As BBG9‐1 remained susceptible to antimicrobials even in the presence of one dominant bacterium (Figure 4). we investigated whether the presence of a wide variety of live intestinal bacteria would influence BBG9‐1 survival. For AMPC, CFDN, and FRPM, the viable BBG9‐1 count was significantly higher at the highest Cell‐Mock concentration (10^9^ CFU/mL) than at the other concentrations tested. For CAM, the viable counts of BBG9‐1 in co‐cultures with 10^8^ and 10^9^ CFU/mL Cell‐Mock were significantly higher than those in co‐cultures with 10^6^ and 10^7^ CFU/mL Cell‐Mock. For LVFX, the viable counts of BBG9‐1 in co‐cultures with 10^8^ CFU/mL Cell‐Mock were significantly higher than those in co‐cultures with 10^6^ and 10^7^ CFU/mL Cell‐Mock (Figure 5). These findings suggest that the presence of multiple dominant bacteria reduces the susceptibility of BBG9‐1 to antimicrobials.

### 3.6 In Vivo Effect of Each Antimicrobial on the Viability of BBG9‐1 in Germ‐Free (GF) Mice

**Figure 6:**
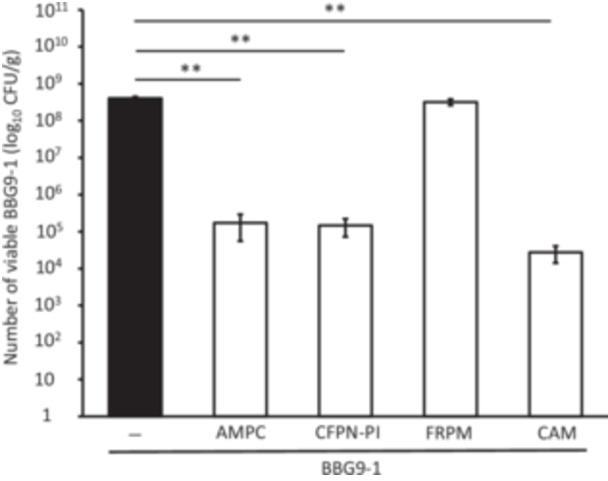
Viable counts of BBG9‐1 in germ‐free (GF) mouse feces. Data (*n* = 3) are presented as mean ± SE. **p < 0.01 by Dunnett's test. AMPC, amoxicillin; BBG9‐1, *Bifidobacterium bifidum* G9‐1; CAM, clarithromycin; CFPN‐PI, cefcapene pivoxil; CFU, colony‐forming unit; FRPM, faropenem.

In vitro, the presence of a wide variety of live intestinal bacteria enabled BBG9‐1 to survive despite antimicrobial exposure. This protective effect was also confirmed in vivo. Initially, we assessed the survival of BBG9‐1 under antimicrobial treatment using GF mice, which lack indigenous gut microbiota. In the absence of antimicrobials, the viable BBG9‐1 count in the feces of GF mice was 2.90 × 10^8^ CFU/g; whereas in the BBG9‐1 + AMPC, CFPN‐PI, and CAM groups, the viable counts of BBG9‐1 were significantly low (approximately 10^5^ CFU/g). Meanwhile, the fecal BBG9‐1 count in the BBG9‐1 + FRPM group was comparable to that in the BBG9‐1 alone group (Figure 6).

### 3.7 In Vivo Effects of Each Antimicrobial on BBG9‐1 Viability and Gut Microbiota in Specific Pathogen‐Free (SPF) Mice

**Figure 7:**
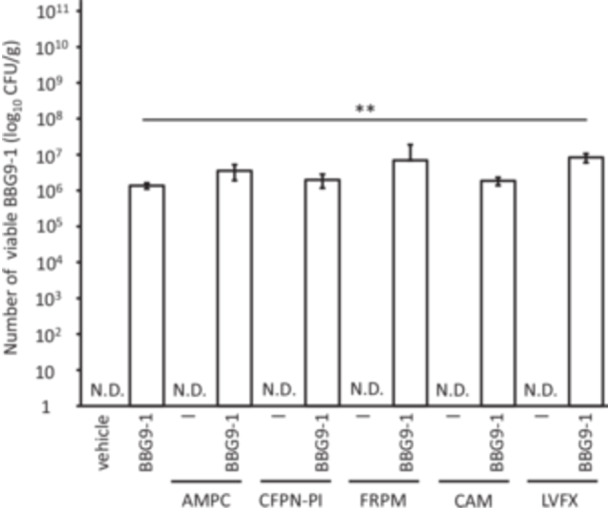
Viable counts of BBG9‐1 in SPF mouse feces. Data (*n* = 7–8) are presented as mean ± SE. **p < 0.01 by Dunnett's test. AMPC, amoxicillin; BBG9‐1, *Bifidobacterium bifidum* G9‐1; CAM, clarithromycin; CFPN‐PI, cefcapene pivoxil; CFU, colony‐forming unit; FRPM, faropenem; LVFX, levofloxacin; N.D., not detected.

**Figure 8:**
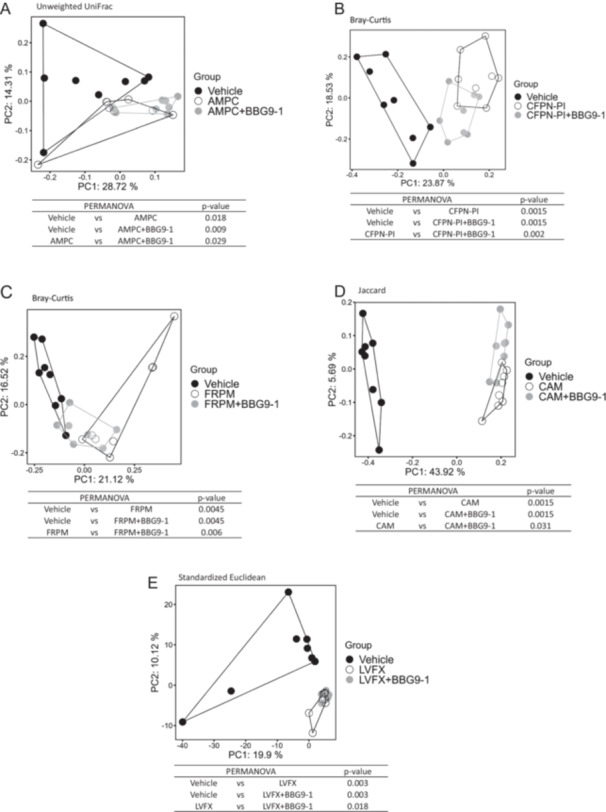
Analysis of the β‐diversity of gut microbiota. Principal coordinate analysis showed clustered communities of fecal microbiota based on different distance metrics: the unweighted UniFrac, Bray–Curtis, Jaccard, and standardized Euclidean (seuclidean) distance between samples. Results of the permutational multivariate analysis of variance for each distance between the fecal microbiota. (A) AMPC treatment. (B) CFPN‐PI treatment. (C) FRPM treatment. (D) CAM treatment. (E) LVFX treatment. AMPC, amoxicillin; BBG9‐1, *Bifidobacterium bifidum* G9‐1; CAM, clarithromycin; CFPN‐PI, cefcapene pivoxil; FRPM, faropenem; LVFX, levofloxacin.

In GF mice, we found that BBG9‐1 survival was markedly reduced under antimicrobial treatment, except in the case of FRPM treatment. Subsequently, we evaluated the survival of BBG9‐1 under antimicrobial exposure in SPF mice. Mice administered BBG9‐1 alone, as well as BBG9‐1 + AMPC, CFPN‐PI, FRPM, and CAM, exhibited similar viable counts of BBG9‐1. Notably, the counts were significantly higher in the BBG9‐1 + LVFX group than in the BBG9‐1 alone group. BBG9‐1 was not detected in any group that received antimicrobials alone (Figure 7). In addition, there was no significant difference in α‐diversity (data not shown). The microbiota composition was significantly different between the vehicle and BBG9‐1 + antimicrobial groups for all antimicrobials. Moreover, the microbiota composition significantly differed between the antimicrobial‐treated and BBG9‐1 + antimicrobial groups (Figure 8). The in vivo studies confirmed that the presence of the intestinal microbiota reduces the extent to which BBG9‐1 is affected by antimicrobials, and further revealed that its influence on the microbial community was maintained.

## 4 Discussion

In this study, we found that in the presence of a wide variety of live intestinal bacteria, the probiotic BBG9‐1 can survive and maintain its ability to influence the gut microbiota both in vitro and in vivo, even when used along with antimicrobials. Although probiotics have been used for a long time, very few studies have elucidated the interactions between antimicrobials and probiotics within the gut environment. MICs for all antimicrobials were low, indicating that BBG9‐1 is highly sensitive to the tested antimicrobials (Table 1). While MIC is measured under pure culture conditions, the vast and diverse bacteria in the gastrointestinal tract may influence this sensitivity to antimicrobials. Therefore, we assessed the effect of antimicrobials on BBG9‐1 in the presence or absence of intestinal bacteria. In the presence of intestinal bacteria, BBG9‐1 viability was higher than that in their absence (Figure 1), suggesting that probiotics that lack inherent antimicrobial resistance may survive in vivo owing to the presence of gut microbiota.

We then assessed the effect of the viability status of the intestinal bacteria on BBG9‐1 viability after treatment with AMPC, FRPM, CAM, and LVFX. The group with viable intestinal bacteria exhibited a higher viable BBG9‐1 count than the group with dead intestinal bacteria (Figure 2). These results suggest that BBG9‐1 is directly affected by antimicrobials in a pure in vitro culture environment, but the presence of viable intestinal bacteria protects it from antimicrobials and allows it to survive in the gut.

We hypothesize that BBG9‐1 was able to survive because the dominant bacteria in the intestine were more affected by the antimicrobials. The intestinal microbiota analysis of SPF mice (data not shown) showed that *L. hamsteri*, with a high relative abundance, was the dominant bacterium, and *Bacteroides*, with a low relative abundance, was the nondominant bacterium. First, we determined the viable counts of dominant *Lactobacillus* and nondominant *Bacteroides* in the feces of SPF mice treated with antimicrobials, and found that the dominant bacterium was more affected by the antimicrobials (Figure 3). Although antimicrobials act on bacterial growth, the dominant bacterium is possibly more affected by them than the nondominant bacterium because of its superior ability to grow. Additionally, drug absorption mostly occurs in the small intestine [18], and the drugs act on bacteria in the small intestine. This could allow BBG9‐1 to circumvent the effect of the antimicrobials, as it can grow only in the large intestine.

We found that the viability of both *Lactobacillus* (dominant) and BBG9‐1 (nondominant) decreased to a similar extent (Figure 4). It was assumed that the effect of the antimicrobials would predominantly be on the dominant bacteria and that the nondominant bacteria would not be affected as much. However, both types of bacteria were strongly affected by the antimicrobials, regardless of the number of bacteria present at the start. Therefore, it is possible that the antimicrobials can affect both types of bacteria without considerable dispersion because of the absence of other competing bacteria. Using Cell‐Mock, a cocktail containing 20 types of human commensal bacteria to simulate intestinal microbiota, we observed that the higher the number of cocultured bacteria, the higher the survival rate of BBG9‐1. These results suggest that the presence of diverse and robust live gut bacteria in vitro can protect BBG9‐1 from antimicrobials, improving its survival.

In this study, we demonstrated that BBG9‐1 had a higher survival rate when the fecal microbiota were alive or in a Cell‐Mock community. However, the underlying mechanisms are not fully understood. As BBG9‐1 does not produce β‐lactamase and is not capable of forming biofilms (data not published), these common mechanisms of antimicrobial resistance are unlikely to be directly involved in the mechanism of resistance. Nevertheless, some coexisting gut bacteria may produce β‐lactamases [19], which may contribute to antibiotic degradation and thereby indirectly protect BBG9‐1 [20]. Additionally, Li et al. demonstrated that *Bifidobacterium breve* forms a symbiotic biofilm with *Akkermansia muciniphila* and *Bacteroides ovatus*, which attenuates the effect of antibiotics on bacteria [21]. On the basis of these results, BBG9‐1 may receive indirect protection from interactions with the surrounding gut microbiota, which may aid in shielding from antimicrobials. These hypotheses merit further investigations, and which may provide important insights on how gut microbiota influences the survival of probiotics under antimicrobial pressure.

Finally, we examined whether the results observed in the in vitro experiments could be recapitulated in vivo. In GF mice, after antimicrobial and BBG9‐1 administration, BBG9‐1 viability significantly decreased, except under FRPM treatment (Figure 6). Meanwhile, in SPF mice, the viable counts of BBG9‐1 in combination with any antimicrobial were comparable to those in the group treated with BBG9‐1 alone. Therefore, in the presence of intestinal bacteria, BBG9‐1 was able to survive in the presence of an antimicrobial, consistent with our in vitro findings. Furthermore, the gut microbiota in SPF mice treated with an antimicrobial and BBG9‐1 was altered compared with that in mice treated with antimicrobials alone. The presence of BBG9‐1 was thought to affect the gut microbiota composition altered by antimicrobial administration. We found that BBG9‐1 can survive in the gastrointestinal tract and influence the gut microbiota even when co‐administered with antimicrobials.

Our study has some limitations. First, the types of antimicrobials that were tested were limited. Second, the efficacy of BBG9‐1 in combination with antimicrobials in humans was not examined, necessitating further research.

In conclusion, in the presence of a wide variety of live intestinal bacteria, the probiotic BBG9‐1 can survive and maintain its ability to influence the gut microbiota both in vitro and in vivo, even when used along with antimicrobials. These findings suggest that BBG9‐1 may be a suitable probiotic candidate for use alongside certain antimicrobial therapies.

## Author Contributions

All authors meet the ICMJE authorship criteria. H.Y. contributed to data analysis, statistical analysis, and manuscript drafting. Y.M. contributed to study conceptualization, study design, data collection, and manuscript revision. Y.T. contributed to the conceptualization of the study, the design of the study, and the manuscript revision. H.O. supervised the study and revised the manuscript. All authors approved the final manuscript as submitted and agreed to be accountable for all aspects of the work.

## Ethics Statement

All animal studies were conducted after approval by the Animal Experimentation Committee of Biofermin Pharmaceutical Co. Ltd. (Approval Numbers: 134‐008 and 137‐014).

## Consent

The authors have nothing to report.

## Conflicts of Interest

The authors declare no conflicts of interest.

## Supporting information

TableS1.

## Data Availability

The data that support the findings of this study are available from the corresponding author upon reasonable request.
