# Supplementary material for: Bifidobacterium bifidum G9‐1 Survives in the Intestinal Environment and Influences the Gut Microbiota Despite the Presence of Antimicrobials
Source: Microbiol Immunol. 2025 Jun 29;69(9):447–56. doi: 10.1111/1348-0421.13230 (PMC12414442; doi:10.1111/1348-0421.13230)
Supplement: Supplementary file 1 — TableS1. [file MIM-69-447-s001.docx]

**Supplementary table**

**Table S1.** Configuration of Cell-Mock-003.

| Bacteria | NBRC number | Gram stain |
| --- | --- | --- |
| *Anaerostipes caccae* | 114412 | Positive |
| *Bifidobacterium longum* | 114370 | Positive |
| *Bifidobacterium longum*subsp.*longum* | 114494 | Positive |
| *Blautia parvula* | 113351^T^ | Positive |
| *Collinsella aerofaciens* | 114504 | Positive |
| *Enterocloster clostridioformis* | 113352 | Positive |
| *Flavonifractor plautii* | 113805 | Positive |
| *Lactobacillus delbrueckii* | 3202 | Positive |
| *Mediterraneibacter gnavus* | 114413 | Positive |
| *Akkermansia muciniphila* | 114322 | Negative |
| *Bacteroides uniformis* | 113350 | Negative |
| *Escherichia coli* | 3301 | Negative |
| *Parabacteroides distasonis* | 113806 | Negative |
| *Streptococcus mutans* | 13955^T^ | Positive |
| *Cutibacterium acnes*subsp. *acnes* | 113869 | Positive |
| *Staphylococcus epidermidis* | 113846 | Positive |
| *Bacillus subtilis* | 13719^T^ | Positive |
| *Pseudomonas putida* | 14164^T^ | Negative |
